# Molecular Mechanisms for Age-Associated Mitochondrial Deficiency in Skeletal Muscle

**DOI:** 10.1155/2012/768304

**Published:** 2012-04-04

**Authors:** Akira Wagatsuma, Kunihiro Sakuma

**Affiliations:** ^1^Graduate School of Information Science and Technology, The University of Tokyo, 7-3-1 Hongo, Bunkyo-ku, Tokyo 113-8656, Japan; ^2^Research Center for Physical Fitness, Sports and Health, Toyohashi University of Technology, 1-1 Hibarigaoka, Tenpaku-cho, Toyohashi 441-8580, Japan

## Abstract

The abundance, morphology, and functional properties of mitochondria decay in skeletal muscle during the process of ageing. Although the precise mechanisms remain to be elucidated, these mechanisms include decreased mitochondrial DNA (mtDNA) repair and mitochondrial biogenesis. Mitochondria possess their own protection system to repair mtDNA damage, which leads to defects of mtDNA-encoded gene expression and respiratory chain complex enzymes. However, mtDNA mutations have shown to be accumulated with age in skeletal muscle. When damaged mitochondria are eliminated by autophagy, mitochondrial biogenesis plays an important role in sustaining energy production and physiological homeostasis. The capacity for mitochondrial biogenesis has shown to decrease with age in skeletal muscle, contributing to progressive mitochondrial deficiency. Understanding how these endogenous systems adapt to altered physiological conditions during the process of ageing will provide a valuable insight into the underlying mechanisms that regulate cellular homeostasis. Here we will summarize the current knowledge about the molecular mechanisms responsible for age-associated mitochondrial deficiency in skeletal muscle. In particular, recent findings on the role of mtDNA repair and mitochondrial biogenesis in maintaining mitochondrial functionality in aged skeletal muscle will be highlighted.

## 1. Introduction

Mitochondria are ubiquitous membrane-bound organelles that are a defining and unique feature of the eukaryotic cell [1]. Mitochondria produce about 90% of the energy necessary for cellular function via oxidative phosphorylation (OXPHOS) [2]. Additionally, mitochondria play central roles in metabolism, signaling, and programmed cell death [3]. Mitochondrial deficiency has been shown in neuromuscular diseases, neurodegenerative diseases, type 2 diabetes, ageing, and sarcopenia [4–8]. During the process of ageing, the abundance, morphology, and functional properties of mitochondria decay in several tissues including skeletal muscle [9–12]. Among all cell types, the skeletal muscle possesses the unique ability to increase metabolic rate nearly 100-fold during the transition form a basal resting state to near-maximal contractile activity [11]. Thus, it is easy to assume that skeletal muscle is profoundly affected by age-associated mitochondrial deficiency. Despite age-associated mitochondrial deficiency being extensively investigated, the precise mechanisms remain to be elucidated. However, these mechanisms include at least decreased mitochondrial DNA (mtDNA) repair, defective mitochondrial removal, and decreased mitochondrial biogenesis, ultimately resulting in decreased function of the whole organelle. The mitochondrial theory of ageing [13] is thought to be one of the most popular theories of ageing [14]. The mitochondrial theory of aging can be summarized as follows: reactive oxygen species (ROS) emanating from mitochondrial OXPHOS are responsible for the accumulation of somatic mtDNA mutations. These mutations are postulated to result in the production of faulty OXPHOS components, leading to the production of still more ROS. Enhanced mitochondrial ROS production and damage can subsequently lead to a “vicious cycle” of exponentially increasing levels of mtDNA damage and oxidative stress in the cell [15]. To prevent deleterious effects in organelle, mtDNA damage is balanced by their own repair system [16]. The changes in the fidelity and efficacy of mtDNA repair may lead to mtDNA damage accumulation during the process of ageing [16]. As damaged mitochondria are eliminated by autophagy (termed mitophagy), mitochondrial biogenesis plays an important role in sustaining energy production and physiological homeostasis in the cell [12]. The capacity for mitochondrial biogenesis has shown to decrease with age, contributing to progressive mitochondrial deficiency [17]. At the molecular level, several transcription factors, nuclear receptors, and coactivators are involved in the activation and regulation of mitochondrial biogenesis. Accumulating evidence suggest that peroxisome proliferator-activated receptor gamma coactivator-1 alpha (PGC-1*α*) plays a key role in mitochondrial biogenesis [1, 3, 18, 19]. PGC-1*α* works in concert with several transcription factors and nuclear receptors to activate expression of a broad set of mitochondrial genes [1]. The activity of PGC-1*α* is regulated by a variety of posttranslational modifications such as phosphorylation and acetylation [1, 3, 18, 20]. These modifications have assumed to be an important mechanism that could regulate PGC-1*α* transcriptional activity [3, 18]. Understanding how these endogenous systems adapt to altered physiological conditions during the process of ageing will provide a valuable insight into the underlying mechanisms that regulate cellular homeostasis [21]. Here we will summarize the current knowledge about the molecular mechanisms responsible for age-associated mitochondrial deficiency in skeletal muscle. In particular, recent findings on the role of mtDNA repair and mitochondrial biogenesis in maintaining mitochondrial functionality in aged skeletal muscle will be highlighted.

## 2. Mitochondrial DNA Mutations in Aged Skeletal Muscle

Numerous studies have shown that mtDNA accumulates oxidative damage in an age-dependent manner in a variety of tissue [22]. The mutations include deletions, point mutations, and duplications that have been found to accumulate in mtDNA in several tissues of various species during the process of ageing [7]. It has been estimated that the proportions of mutated mtDNA in ageing human tissues rarely exceed 1% [22]. How this apparent low level mutation can be functionally relevant in ageing continues to be topics of vigorous debate [22]. In the case of aged skeletal muscle, mtDNA deletion mutations are at low abundance (<0.1%) when calculated against the total mitochondrial pool in tissue homogenates [23]. When, however, discrete myofibers are analyzed, the abundance of mtDNA deletion mutations is inversely proportional to the number of myofibers analyzed [24]. In support of this, mtDNA deletion mutations are not distributed homogeneously throughout a tissue, but amplified focally within a subset of myofibers, appearing as a segmental pattern along the length of myofibers and as a mosaic distribution between myofibers [25]. Mitochondria possess their own DNA repair system to protect cells from mutations. Although the DNA repair mechanisms in mitochondria are not as well known as those taking place in the nucleus, the knowledge on mtDNA repair functions is increasing [26]. It was reported an expanded range of mitochondrial DNA repair systems such as base excision repair (BER) including single-nucleotide and long-patch BER, double-strand break repair (homologous recombination and nonhomologous end-joining), and direct repair [16]. Here we provide information on mtDNA mutations in aged skeletal muscle and mtDNA repair system. We discuss the ageing effects on mtDNA repair activity in skeletal muscle.

Mitochondria possess their own DNA. Each mitochondrion consists of 16,569 base pairs which encode 37 genes encoding 13 genes that are required for OXPHOS, 22 transfer RNAs, and 2 ribosomal RNAs [27]. Despite the fact that mtDNA comprises only 1–3% of genetic material in animal cells, several lines of evidence suggest that its contribution to cellular physiology could be much greater than would be expected from its size alone. For instance, (a) it mutates at higher rates than nuclear DNA; (b) it encodes either polypeptides of electron transport system (ETS) or components required for their synthesis such as tRNAs and rRNAs, and therefore, any coding mutations in mtDNA will affect the ETS as a whole; this could affect both the assembly and function of the products of numerous nuclear genes in ETS complexes; (c) defects in the ETS can have pleiotropic effects because they affect cellular energetics as a whole [28].

Abnormalities of mitochondrial ETS enzymatic activities have been identified using a histochemical method. Activities of two complexes of the ETS are commonly analyzed: complex II (succinate dehydrogenase; SDH) and complex IV (cytochrome c oxidase; COX). Abnormal myofibers are characterized by negative for COX and hyperreactive for SDH [25]. These fibers have been termed ‘‘ragged-red” fibers (RRFs), which display a granular, mottled red appearance with the modified Gomori trichrome stain [29]. RRF was initially characterized in human mitochondrial myopathy diseases that are neuromuscular disorders associated with defects of the mitochondrial genome [30]. The frequency of RRFs in old humans (61 to 77 years of age) was much higher than that in young humans (21 to 31 years of age) (0.33 *versus* 0.02%) [31]. In animal, the frequency of myofibers exhibiting ETS abnormalities in old (34 years) and young adult (11 years) rhesus monkeys was 2.41 and 0.16%, respectively [32]. Interestingly, it could be estimated, however, that up to 60% of the myofibers display ETS abnormalities in the old animal compared to 4% in the young adult animal, when extrapolating the data to the entire length of the myofiber [32].

The ETS abnormalities observed in aged skeletal muscle appear to correlate closely with mtDNA deletion mutations. It has been reported that mtDNA 4977-bp deletion correlates with respiratory chain enzyme activities in ageing human skeletal muscles [33]. This is the most frequent and best characterized age-associated mtDNA deletion mutation, called the “common deletion.” This deletion is especially frequent in well-differentiated cells with a low mitotic rate, such as brain and muscle cells [34]. Cao et al. [35] determined the mitochondrial genotype of aged rat myofibers exhibiting ETS abnormalities using histochemical analysis and laser-capture microdissection combined with PCR. Large mtDNA deletion (4.4–9.7 kb) was detected in all ETS abnormal myofibers. The deleted mtDNA genomes were detected only in the regions of the myofibers with ETS abnormalities; adjacent phenotypically normal portions of the same fiber contained wild-type mtDNA. Additionally, identical mtDNA deletion mutations were found within different sections of the same abnormal region. They suggest that large deletion mutations are associated with ETS abnormalities in aged rat skeletal muscle and that a clonal expansion of deleted mitochondrial genome occurs in ETS abnormal regions of myofibers. Similarly, Herbst et al. [36] reported that aged myofibers possessed segmental, clonal intracellular expansions of unique somatically derived mtDNA deletion mutations. When the mutation abundance surpassed 90% of the total mitochondrial genomes, the myofiber lost COX activity and exhibited an increase in SDH activity. These findings support the idea that mutated mtDNA may have replicative advantages over normal DNA, resulting, in turn, in preferential propagation (clonal expansion) of defective mitochondria. Augmented replication of mutated mtDNA remains, however, unexplained. It is hypothesized that deleted DNA replicates faster due to its shorter length, although this explanation cannot be applied to DNA with point mutations [37]. Besides the mitochondrial enzymatic abnormalities, some myofibers displayed abnormal morphology such as fibers splitting, atrophy, and breakage. Therefore, it is speculated that the somatic generation and subsequent intracellular accumulation of mtDNA deletion mutations may contribute to the age-dependent loss of myofibers and sarcopenia [36].

Besides deletion mutations, point mutations, and duplications also occur in mtDNA of aged skeletal muscle. Zhang et al. [38] reported the age-related mutation A to G at nucleotide potion 3243 on mtDNA in skeletal muscle. Munscher et al. [39] reported the A8344G transition mutation in the tRNA^Lys^ gene of mtDNA, characteristic for the maternally inherited MERRF syndrome (myoclonic epilepsy with ragged red fibers) in extraocular muscle from individuals aged 67–89 years old. The amount of mutated from total mtDNA was estimated in extraocular muscle of 2 individuals of 74 and 89 years to 2.0 and 2.4%, respectively. Fayet et al. [40] reported that high levels of clonally expanded point mutations were identified in eight cytochrome-c-oxidase-deficient myofibres from individuals, without muscle disease, aged 69–82 years but in none of the normal ones. They included the previously described pathogenic tRNA^Leu^ (UUR) A3243G and tRNA^Lys^ A8344G mutations and three original mutations: tRNA^Met^ T4460C, tRNA^Met^ G4421A, and a 3-bp deletion in the tRNA^Leu^ (UUR) gene. Wang et al. [41] reported that muscle-specific mutations accumulate with ageing in critical human mtDNA control sites for replication. Most of 26 individuals 53 to 92 years old, without a known history of neuromuscular disease, exhibited at mtDNA replication control sites in skeletal muscle an accumulation of two new point mutations, that is, A189G and T408A, which were absent or marginally present in 19 individuals younger than 34 years. These two mutations were not found in fibroblasts from 22 subjects 64 to 101 years of age (T408A), or were present only in three subjects in very low amounts (A189G). They suggest that the striking tissue specificity of the skeletal muscle mtDNA mutations detected and their mapping at critical sites for mtDNA replication strongly point to the involvement of a specific mutagenic machinery and to the functional relevance of these mutations. Lee [42] detected a −260 bp (type I) and a −200 bp (type II) tandem duplications in the mtDNA of muscle biopsies from aged individuals. Only one 70-year-old subject was found to harbour the type I and 28 out of 58 subjects had type II duplication. About 90% of the subjects harbouring the duplicated mtDNAs also had the 4,977 bp deletion. Moreover, the incidence and quantity of the type II duplication were found to increase with age. The proportion of the type II duplicated mtDNA in the muscle of a 71-year-old individual was 3.1% while that of a 55-year-old individual was only 0.78%. They suggest that the tandem duplications occur alone or with mtDNA deletions in skeletal muscle in an age-dependent manner, and thereby cause synergistic deleterious effects on mitochondrial respiratory chain functions in human ageing. 

## 3. Mitochondrial DNA Repair Pathway

ROS are generally regarded as being detrimental to cellular function by modifying the structure and function of proteins, lipids, and nucleic acid. mtDNA may be more susceptible to damage by ROS than nuclear DNA, because of the proximity of mtDNA to the source of ROS, altered chromatin packaging due to the lack of histones, the lack of introns, and the compactness of its genetic information, inefficient DNA repair systems, and asymmetry of mtDNA replication [43–45]. Consequently, mutational damage accumulates more rapidly in mtDNA compared to nuclear DNA, leading to dysfunctional and defects in the respiratory chain [40]. Therefore, DNA repair is thought to be an important mechanism for maintenance of genomic integrity. Currently, it is of great interest to understand whether the mtDNA repair mechanisms, particularly base excision repair, play an important role in the aging process and age-associated diseases. Mitochondrial BER (mtBER) includes several distinct steps [26]. The first step in single-nucleotide mtBER is the elimination of the damaged nucleotide. The abnormal base is removed by a DNA glycosylase, which recognizes the modified or inappropriate base and cleaves the N-glycosidic bond, creating an abasic site [46]. Human mitochondria possess and several DNA glycosylases such as uracil-DNA glycosylase, 8-oxoguanine DNA glycosylase, MutY homolog DNA glycosylase [47]. One of the most extensively investigated mitochondrial DNA glycosylases is 8-oxoguanine DNA glycosylase (OGG1). OGG1 is a DNA glycosylase with AP lyase activity that recognizes and cleaves 7,8-dihydro-8-oxoguanine (8-oxoG) from DNA [48]. The 8-oxoG DNA lesion, which can mispair with adenine and thereby introduce GC→TA transversions during replication [49], is one of the most abundant oxidative lesions which accumulates in the mitochondria with age [50]. The remaining abasic nucleotide (AP site for apurinic and apyrimidinic site) is eliminated by a AP endonuclease (APE), which has two major enzymatic functions: (a) cleavage of the phosphodiester backbone 5′ to AP sites, generating 3′-OH substrates for DNA polymerases; (b) 3′-diesterase activity specific for the 3′-dRP products generated by *β*-elimination by bifunctional glycosylases, again creating 3′-OH substrates for DNA polymerases [46]. APE1 has been described to be the main AP endonuclease in mammalian cells [46]. The next step requires DNA polymerase-mediated de novo insertion of a single nucleotide [16]. Mammalian mitochondria posses a single DNA polymerase, polymerase *γ* [51]. The final step requires the ligation of the remaining single-strand nick, which is catalyzed by a DNA ligase [46]. The human DNA ligase III gene encodes both nuclear and mitochondrial form of ligase [52]. For details on other mtDNA repair pathways, please refer to some excellent reviews [16, 26].

## 4. Effects of Ageing on mtBER Activity in Skeletal Muscle

It has been shown that the levels of overall mtBER activity in rat cerebral cortices gradually are decreased with age, reaching 80% lower activity in 30-month-old rats, compared with 17-day-old embryos [53]. The decrease in overall mtBER activity with age appears to attribute to the decreased expression of repair enzymes such as OGG1 and DNA polymerase *γ* [53]. Another study showed a significant age-dependent decrease in incision activities of all three glycosylases including uracil-DNA glycosylase, 8-oxoguanine DNA glycosylase, and endonuclease III homologue in the mitochondria of all brain regions [54]. In skeletal muscle, several studies have investigated the activity or expression of specific mtBER-related enzymes in aged animals [55–58]. For example, there were no changes in the expression levels of DNA polymerase *γ* (POL*γ*A) and DNA polymerase *γ* accessory subunit (POL*γ*B) transcripts in skeletal muscle of young (21 to 27 years of age) and older humans (65 to 75 years of age) [55]. The expression levels of OGG1 protein were slightly increased in fast-twitch muscle from 36-month-old compared to 6-month-old rats [56], suggesting that the level of ROS production reaches a critical threshold to produce oxidative stress since this enzyme is considered to be a useful biomarker for assessing oxidative stress [59]. This may be a compensatory response to oxidative stress to attenuate mtDNA damage. This hypothesis is partially supported in previous studies. When a vector containing a mitochondrial transport sequence upstream of the sequence for human OGG1 was transfected into oligodendrocytes which are extremely sensitive to oxidative stress, it promoted an increase in mtDNA repair and a decrease in caspase-9-dependent apoptosis after exposure of the cells to menadione, a soluble form of vitamin K, which is often used as a generator of ROS [60]. When the expression of the human OGG1 protein was targeted to the mitochondria in HeLa cells, their capacity to repair mtDNA oxidative damages that are induced by various agents was enhanced, leading to the increase in cell survival under oxidative stress [61]. To date, few have measured the impact of ageing overall mtBER capacity in aged skeletal muscle. It was, however, recently reported that mtBER activity in skeletal muscle is regulated in an age-dependent manner. Szczesny et al. [62] investigated age- and tissue-dependent changes in DNA BER of oxidative lesions in mitochondrial extracts by measuring single-nucleotide (SN)-BER and long-patch (LP)-BER activities in several tissues isolated from 4-, 10- and 20-month-old mice. The mtBER activity was determined by analyzing the total repair synthesis using duplex oligonucleotides containing uracil or tetrahydrofuran, specific lesions for SN- and LP-BER, respectively. Age-dependent SN-BER activity was increased in liver, kidney, heart, and quadriceps (fast-twitch muscle) but not in pectoralis (slow-twitch muscle). LP-BER activity was also increased in liver, kidney, and heart but not in quadriceps and pectoralis. APE activity, which is a key component of BER and is believed to be rate limiting in the repair of oxidative DNA damage [63], was decreased in both skeletal muscles with age. Besides mtBER activity, the expression levels of antioxidant enzymes including manganese superoxide dismutase, copper- and zinc-containing superoxide dismutase, and catalase were also lower in skeletal muscle than in liver, kidney, and heart irrespective of age. They hypothesize that relatively low levels of mtBER activity and antioxidant enzymes in skeletal muscle may contribute to age-associated mitochondrial deficiency.

 As above mentioned, large deletions in mtDNA are frequently observed in aged skeletal muscle. It has been proposed that the generation of these deletions result from double-strand breaks in mtDNA during repair of damaged mtDNA rather than during replication [64]. Double-strand breaks in mtDNA can be repaired by homologous recombination or nonhomologous end-joining mechanisms, as it occurs in the nucleus [16, 26]. Evidence for both homologous recombination and nonhomologous end-joining processes in mammalian mitochondria has been reported. For example, mitochondrial protein extracts from normal and immortalized mammalian somatic cells catalyzed homologous recombination of plasmid DNA substrates [65]. When incubating linearized plasmid DNA with mitochondrial protein extracts from rodent liver, DNA end-joining activity was observed [66]. Further, it was recently showed that human Rad51, which is required for homologous recombination, localizes also to mitochondria in human cells and could potentially be involved in organelle double-strand break repair [67]. However, many other enzymes associated with these mtDNA repair mechanisms have not been identified in the animal mitochondria [68]. It could be speculated that mitochondrial recombinational DNA repair in mammalian cells may depend on enzymes involved in other DNA repair pathways [26]. The significance of these mtDNA repair mechanisms is still a developing area for future investigation. Thus, although it remains unclear to what extent these mtDNA repair systems contribute to attenuate age-related accumulation of mtDNA mutations in skeletal muscle, it may be due to the decreased capacity for mtDNA repair and/or insufficient mtDNA repair mechanisms.

## 5. Transcriptional Control of Mitochondrial Biogenesis

During the last decade or two, there was much progress in understanding transcriptional regulatory mechanisms governing mitochondria biogenesis. Mitochondrial biogenesis is a complex process that requires the synthesis, import, and incorporation of proteins and lipids to the existing mitochondrial reticulum as well as replication of the mtDNA [3]. The mitochondrial proteome comprises approximately 1,500 different proteins [69]. More than 98% of the total protein complement of mitochondria is encoded by the nuclear genome although the organelle has their own DNA [18]. To maintain mitochondrial functionality, mitochondrial biogenesis requires a coordination of expression of two genomes and therefore crosstalk between the nucleus and mitochondria [18]. It is now widely accepted that PGC-1*α* plays a central role in a regulatory network governing the transcriptional control of mitochondrial biogenesis [1, 3, 18, 19]. PGC-1*α* is known to work in concert with a wide variety of interacting partners, which are transcription factors and nuclear receptors [1, 3]. Here we provide some information on the transcriptional events involved in mitochondrial biogenesis. For more complete details, excellent review articles are already available on this subject [1, 3, 18, 70]. We discuss the underlying mechanisms that regulate mitochondrial biogenesis in aged skeletal muscle.

PGC-1*α* was discovered in a yeast two-hybrid screen for brown adipose-specific factors that interact with the nuclear receptor PPAR*γ* and are dramatically induced by exposure to cold in brown fat and skeletal muscle [71]. Subsequently, two additional PGC-1 family members were identified, PGC-1-related coactivator (PRC) [72], and PGC-1*β* [73]. Gain-of-function and loss-of-function studies have revealed the central roles of PGC-1*α*, PRC, and PGC-1*β* in mitochondrial gene expression and biogenesis. Forced expression of PGC-1*α* or PGC-1*β* in C2C12 cells resulted in increased mitochondrial biogenesis and oxygen consumption [74, 75]. PRC overexpressing myotubes showed an elevated fatty acid oxidation and increased expression of mitochondrial genes [76]. Skeletal muscle-specific PGC-1*α* or PGC-1*β* transgenic mice exhibited increased mtDNA amount, mitochondrial content, mitochondrial enzyme activity, increased expression of mitochondrial genes, and enhanced exercise performance [77–79]. PGC-1 family coactivators appear to be involved in a myofiber-type conversion. PGC-1*α* or PRC drives formation of type I and IIA myofibers [76, 77, 79], which contain abundant mitochondria [80–82], whereas PGC-1*β* promotes formation of type IIX myofibers [78], which are highly oxidative but have fast twitch characteristics [80–82]. On the other hand, PGC-1*α* deficiency mice exhibited that mitochondrial number and respiratory capacity is diminished in skeletal muscle, leading to reduced muscle performance and exercise capacity [83]. Skeletal-muscle-specific PGC-1*α* knockout mice showed decreased expression of mitochondrial genes, a shift from oxidative type I and IIa toward type IIx and IIb myofibers and reduced endurance capacity [84]. Mice lacking PGC-1*β* showed a reduced numbers of mitochondria, decreased respiration function, and decreased expression of mitochondrial genes [85]. Short hairpin RNA-mediated silencing of PRC resulted in a severe respiratory chain deficiency associated with the proliferation of aberrant mitochondria in U20S cells [86]. Recently, mice with total PGC-1 deficiency (PGC-1*α* ^−/−^ *β* ^f/f/MLC-Cre^ mice) in skeletal muscle have been characterized [87]. PGC-1*α* ^−/−^ *β* ^f/f/MLC-Cre^ mice exhibited a dramatic reduction in exercise performance compared to single PGC-1*α* or PGC-1*β*-deficient mice and wild-type mice. Additionally, PGC-1*α* ^−/−^ *β* ^f/f/MLC-Cre^ mice showed mitochondrial structural derangements consistent with fusion/fission and biogenic defects concomitantly with no reduction of the proportion of oxidative types I and IIa myofibers [87].

The three coactivators regulate expression of a broad set of mitochondrial genes and promote mitochondrial biogenesis [88]. Because these coactivators lack DNA-binding activity [89, 90], PGC-1 family coactivators exert their effects through interactions with transcription factors and nuclear receptors bound to specific DNA elements in the promoter region of genes. For example, nuclear respiratory factor-1 (NRF-1) and NRF-2 (GA-binding protein; GABP) were the first regulatory factors implicated in the global expression of multiple mitochondrial functions in vertebrates [19]. Both NRF-1 and NRF-2 link to the transcriptional control of nuclear and mitochondrial genes involved in OXPHOS, electron transport (complex I–V), mtDNA transcription/replication, heme biosynthesis, protein import/assembly, ion channels, shuttles, and translation [70]. As to mtDNA transcription/replication, NRF-1 or NRF-2 contribute to expression of nuclear encoded genes involved in mtDNA transcription/replication machineries including mitochondrial transcription factor A (TFAM), mitochondrial transcription factor B1 or B2 (TFB1M or TFB2M), and mitochondrial RNA polymerase (POLRMT), and mitochondrial transcription termination factor (MTERF), mitochondrial DNA helicase (TWINKLE), single-stranded DNA-binding protein (mtSSB), and POL*γ*B [91–93] but not POL*γ*A and MTERF3 [93]. Cotransfection of PGC-1*α* along with NRF-1 increases the transcription of TFAM, a direct regulator of mtDNA replication/transcription, to a greater extent compared to the expression of PGC-1*α* alone [74]. Interestingly, skeletal-muscle-specific knockout mice lacking GABP*α*, the DNA-binding subunit of GABP, exhibited no overt muscle defects, with normal distribution and appearance of mitochondria [94, 95], suggesting that GABP might not be essential for muscle mitochondrial biogenesis and that a compensatory mechanism might exist to maintain mitochondrial gene expression.

Peroxisome proliferator-activated receptors (PPARs) were discovered as ligand-activated transcription factors and are classified as members of the steroid hormone receptor superfamily [96]. To date, three related PPAR isoforms have been identified including PPAR*α*, PPAR*β*/*δ*, and PPAR*γ* [97]. PPARs act as heterodimers with retinoid X receptors (RXRs) to regulate a broad set of genes involved in lipid uptake, storage, and metabolism [98]. Skeletal muscles from PPAR*α* knockout mice exhibited only minor changes in fatty acid homeostasis and that neither constitutive nor inducible mRNA expression of known PPAR*α* target genes is negatively affected by its absence [99]. The highly selective PPAR*δ* agonist GW742 increased fatty acid oxidation and induced expression of several lipid regulatory genes in both primary human satellite cells and L6 myogenic myotubes. The responses are similar to those elicited by the PPAR*α* agonist GW647, whereas the PPAR*γ* agonist, GW929, did not affect this gene expression [99]. Thus, it is likely that redundancy in the functions of PPARs *α* and *δ* as transcriptional regulators of fatty acid homeostasis and high levels of the *δ*-subtype expressed in skeletal muscle can compensate for deficiency of PPAR*α* [99]. Independent expression of either PPAR*α* or PGC-1 leads to a modest increase in the expression of medium-chain acyl-CoA dehydrogenase (MCAD), long-chain acyl-CoA dehydrogenase (LCAD), and carnitine palmitoyltransferase I (CPT I), whereas coexpression of both PPAR*α* and PGC-1 leads to a marked coordinate increase in the expression of the mitochondrial fatty acid oxidation enzymes in the absence or presence of PPAR*α* ligand [100].

Estrogen-related receptor *α* (ERR*α*) is an orphan member of the superfamily of nuclear hormone receptors. ERR*α* was initially isolated based on its sequence homology to the estrogen receptor [101] but is not activated by classic estrogens [102]. ERR*α* has been shown to be a transcriptional regulator of MCAD gene [103], which is one of three nuclearly encoded proteins mediating the initial step in mitochondrial *β*-oxidation of fatty acids [104]. ERR*α* null mice showed a decreased fat mass and are resistant to a high-fat-diet-induced obesity [105]. In C2C12 myogenic cells, ERR*α* overexpression accelerated differentiation with an increased mitochondrial mass, whereas ERR*α* antagonist, XCT790 treatment delays myogenesis and resulted in myotubes with fewer mitochondria and disorganized sarcomeres [106]. Similarly, ERR^−/−^ primary myogenic cells showed delayed myogenesis, resulting in structurally immature myotubes with reduced sarcomeric assembly and mitochondrial function [106]. When ERR*α* and PGC-1*α* are cotransfected, they synergistically enhance the promoter activity of medium-chain acyl-CoA dehydrogenase gene compared with either ERR*α* or PGC-1*α* [107]. PGC-1*β* stimulates ERR*α*-mediated transcription in a dose-dependent manner by means of the ERR-responsive element from the MCAD gene promoter [108].

## 6. Effects of Ageing on Mitochondrial Biogenesis in Skeletal Muscle

 Overall, exercise and muscle regeneration promote mitochondrial biogenesis, whereas physical inactivity and ageing decrease it [109–115], suggesting that mitochondrial biogenesis is tuned to meet cell-specific energetic, metabolic, and signal demands. Although ageing decreases the expression of mitochondrial genes involved in OXPHOS, electron transport/ATP synthase, which are encoded in nuclear and mitochondrial genome in skeletal muscle [50, 116–118], the molecular mechanisms are not yet well understood. We discuss several possibilities that may account for the age-related downregulation of mitochondrial genes in skeletal muscle. This may be the result of reduced mtDNA copy number with age. Transcripts corresponding to the mitochondrial genome are undoubtedly influenced by decreasing mtDNA copy number [118, 119]. Consequently, the expression of the proteins encoded by mitochondrial genome would logically be expected to be influenced by the availability of the mRNA template. Indeed, substantial mtDNA depletion occurred with decreased expression in mitochondria-encoded COX subunits I and III in aged skeletal muscles [119]. Such a decline paralleled reduced COX enzyme activity [119]. More important, however, is that COX transcript levels relative to mtDNA were comparable in young and old animals. This indicates that reduced mtDNA copy number has no major effects on mitochondrial encoded transcript levels [119]. Thus, reduced mtDNA availability may not be a limiting factor of mitochondrial gene expression in aged skeletal muscle. It seems unlikely that age-related reductions of mtDNA may be due to downregulation of several factors essential for mtDNA replication. There was no effect of age on expression of TFAM, NRF-1, POL*γ*A, POL*γ*B, RNase mitochondrial RNA processing (RNase MRP), POLRMT, and mtSSB transcript levels [50, 116–118], suggesting the possibility that mtDNA replication machinery may be preserved during the process of ageing. Additionally, TFAM protein expression was moderately increased in skeletal muscle of aged animals [56]. This may be indicative of a compensatory attempt to attenuate the decay in mitochondrial enzyme activity and functional mtDNA content in senescent skeletal muscle [120]. 

Decreased mitochondrial biogenesis with age may be related to the changes in expression of PGC-1 family coactivators. For example, PGC-1*β* expression was reduced in skeletal muscle from elderly human [121]. We observed decreased expression level of PGC-1*β* transcript in mouse skeletal muscle in an age-dependent manner (our unpublished data). The expression of PGC-1*β* is investigated in an experimental murine model of accelerated ageing, senescence-accelerated prone mice (SAMP) and senescence-accelerated resistant mice (SAMR) [122]. SAMR series normally age, whereas SAMP series exhibit an accelerated aging with a number of common characteristics, such as a moderate-to-severe degree of loss of activity, hair loss and lack of glossiness, periophthalmic lesions, increased lordokyphosis, and early death [123]. Consistent with naturally aged human and mouse, SAMP showed a decrease in PGC-1*β* expression compared to SAMR. In agreement with the proposed role of PGC-1*β* function as a coactivator of ERR*α*, the expression of the ERR*α* target gene MCAD was strongly suppressed in SAMP with the reduction in ERR*α* DNA-binding activity [122]. Additionally, decreased PPAR DNA-binding activity, possibly through enhanced transcriptional repressor, chicken ovalbumin upstream promoter transcription factor II (COUP-TF-II), was observed in SAMP. The enhanced expression of COUP-TF-II has shown to reduce the expression of the PPAR*α*-target gene, acyl-CoA oxidase, which cods for a key enzyme involved in *β*-oxidation [124]. Therefore, fatty acid oxidation is reduced or impaired in SAMP. Unlike PGC-1*β*, conflicting results were observed in previous studies with respect to age-related changes in PGC-1*α* expression. COX activity was decreased concomitantly with decreased expression of PGC-1*α* protein in old (36-months-old) compared to young (6-months-old) rats [56]. Similarly, decreased expression of PGC-1*α* mRNA or protein was observed in skeletal muscle from old human [121], mouse [125] and rat [126]. In contrast, PGC-1*α* mRNA or protein expression remained unchanged with age in human, rat, and mouse skeletal muscles despite a downregulation of mitochondrial genes [117, 118, 122, 127]. We also observed no change in PGC-1*α* mRNA expression among young, middle-aged, and old animals (our unpublished data). These data suggest that age-related decreased mitochondrial biogenesis may not necessarily parallel decreased expression level of PGC-1*α*.

Several lines of evidence suggest that the activity of PGC-1*α* is regulated by a variety of posttranslational modifications, which have assumed to be an important mechanism that could regulate PGC-1*α* transcriptional activity [3, 18]. Multiple signaling pathways have been shown to regulate PGC-1*α* activity. For example, PGC-1*α* has a very short half-life of about 2-3 hours [128], and the mitogen-activated protein kinase (MAPK) p38 phosphorylates, stabilizes PGC-1*α* protein and enhances PGC-1*α* activity [129, 130]. AMP-activated protein kinase (AMPK), which is activated by an increase in intracellular AMP/ATP ratio, has been involved in mitochondrial biogenesis [131]. It was proposed that direct phosphorylations of the PGC-1*α* protein at Thr 177 and Ser 538 are required for the PGC-1*α*-dependent induction of the PGC-1*α* promoter [132]. AMPK can be activated chemically with AICAR (5′-aminoimidazole-4-carboxamide-1-*β*-D-ribofuranoside), which is taken up by cells and phosphorylated by adenosine kinase to form ZMP (5′-aminoimidazole-4-carboxamide-1-*β*-D-ribofuranoside monophosphate) that mimics the effect of AMP [133]. Treatment of mice with AICAR increased expression of mitochondrial proteins and enhanced exercise endurance [134]. AMPK activation via AICAR treatment in C2C12 cells induced PGC-1*α* mRNA expression and transcriptionally activates the PGC-1*α* promoter [135]. When rodents were treated with AICAR, it promoted mitochondrial biogenesis and fatty acid oxidation [136, 137]. Knockout mice lacking both AMPK *β*1 and *β*2 isoforms in skeletal muscle had reduced mitochondrial content and showed drastically impaired capacity for treadmill running [138]. When transgenic mice expressing a dominant-negative mutant of AMPK in muscle were treated with *β*-guanidinopropionic acid (*β*-GPA), a creatine analog, which leads to similar reductions in the intramuscular ATP/AMP ratio and phosphocreatine concentrations, they exhibited abrogated *β*-GPA-induced increases in the mitochondrial content [139]. Interestingly, p38 MAPK and AMPK activation levels in skeletal muscle under steady-state conditions were generally similar between young and old animals, despite the age-related variations in mitochondrial volume [140]. However, the activation of AMPK or p38 MAPK in response to AICAR, exercise, and muscle contraction was blunted in aged skeletal muscle [140, 141]. Therefore, their potential role in regulating PGC-1*α* transcriptional activity may be impaired in aged skeletal muscle. The signaling deficit may, in part at least, account for reduced PGC-1*α*-mediated mitochondrial biogenesis during the process of ageing. Besides phosphorylation, it has been shown that PGC-1*α* activity is potentiated by arginine methylation by arginine methyltransferase 1 (PRMT1) [142]. PRMT1 methylates PGC-1*α* in the C-terminal region and enhances its transcriptional activity. Short interfering RNA-mediated silencing of PRMT1 caused decreases in the expression of PGC-1*α*-induced cytochrome c and ERR*α* genes. Since few studies report a possible role of PRMT1 in aged skeletal muscle, it remains unclear to what extent PRMT1-mediated PGC-1*α* activity contributes to mitochondrial biogenesis. 

PGC-1*α* activity is also modulated by its acetylation state by the deacetylase, SIRT1 (silent mating type-information regulation 2 homolog 1) and the acetyltransferase, GCN5 (general control nonrepressed protein 5) [143]. SIRT1, which is a NAD^+^-dependent deacetylase, deacetylates PGC-1*α* protein and activates PGC-1*α* activity [143]. AMPK acts as the prime initial sensor that translates low nutrient availability or increased energy demand into SIRT1-dependent deacetylation of PGC-1*α* [144]. SIRT1 was bound to PGC-1*α*-targeted gene promoters and relative occupancy at these sites could be significantly enhanced when overexpressing SIRT1 in C2C12 myotubes [145]. The SIRT1 activator SRT1720 also led to a decrease in PGC-1*α* acetylation, and an increase in the expression of PGC-1*α* targets involved in fatty acid oxidation in mouse skeletal muscle [146]. Conversely, knockdown of SIRT1 or the SIRT1 inhibitor, nicotinamide induced PGC-1*α* protein acetylation, decreases expression of PGC-1*α* target genes, and reduced fatty acid oxidation in myotubes [145]. In aged skeletal muscle, SIRT activity was decreased possibly though a decrease in NAD^+^ content, which could be due to reduced expression of NAD^+^ biosynthetic enzyme, NAMPT (nicotinamide phosphoribosyltransferase) [147]. Thus, age-related decreased SIRT1 activity may affect PGC-1*α*-mediated mitochondrial biogenesis. It should be noted, however, that overexpression of SIRT1 in rodent skeletal muscle was shown to downregulate several genes involved in mitochondrial biogenesis despite increased SIRT1 activity [148]. Recently, mice lacking skeletal muscle SIRT1 activity did not impede exercise-induced deacetylation of PGC-1*α* and mitochondrial biogenesis [149]. These results suggest that the role of SIRT1 in the activation of PGC-1*α* mediated transcription appears to be more complex than originally hypothesized. On the other hand, PGC-1*α* is directly acetylated by GCN5, resulting in a transcriptionally inactive protein that relocalizes from promoter regions to nuclear foci [150]. Knockdown of SRC-3 (steroid receptor coactivator-3) in C2C12 cells reduced the expression of GCN5, decreased the acetylation of PGC-1*α*, and increased the expression of PGC-1*α*-regulated mitochondrial genes [151]. PGC-1*β* is also acetylated by GCN5. GCN5 strongly interacted with PGC-1*β* and repressed its transcriptional activity associated with transcription factors such as ERR*α* and NRF-1 [152]. Besides GCN5, two well-established histone acetyltransferases, SRC-1 and CREB-binding protein (CBP)/p300, which have been shown to be strong enhancers of PGC-1*α* activity without acetylating PGC-1*α* by them [89]. This contribution may be not expected since SRC-1 and CBP/p300 proteins were decreased with age in rat motoneurons of the spinal nucleus of the bulbocavernosus [153].

PGC-1*α* activity may be negatively regulated by several factors. Akt/PKB directly phosphorylates PGC-1*α* and prevents the recruitment of PGC-1*α* to the cognate promoters, impairing its ability to promote gluconeogenesis and fatty acid oxidation [154]. Age-related hyperphosphorylation of Akt at Thr 308 and Ser 473 were observed in rat skeletal muscle [155]. Phosphorylation of these two residues is necessary for full activation of Akt kinase activity [156]. It should be noted, however, that age-related Akt hyper-phosphorylation did not result in increased phosphorylation of the Akt downstream molecules such as glycogen synthase kinase3*β* (GSK3*β*) [155]. GSK3*β* also targets PGC-1*α* for nuclear proteasomal degradation and regulates PGC-1*α* activity [157]. In aged skeletal muscle, phosphorylation level of GSK3*β* on Ser 9, which is necessary for GSK3*β* inactivation [158], is decreased with age [159], suggesting that GSK3*β* activity is increased. Thus, age-related change in Akt-GSK3*β* signaling may affect PGC-1*α* turnover and thereby mitochondrial biogenesis. The human receptor-interacting protein 140 (RIP140) was identified as a coactivator for a chimeric estrogen receptor [160]. RIP140 is highly expressed in metabolic tissues such as skeletal muscle [161]. RIP140 has been proposed as suppressor of mitochondrial biogenesis and oxidative metabolism in mammalian cells [162]. The possible role of RIP140 has been confirmed by genetically modified mice. RIP140 null mice exhibited an increase in size and quantity of mitochondria, amount of mtDNA, and number of oxidative fibers in fast-twitch muscle concomitantly with upregulation of genes involved in OXPHOS, fatty-acid oxidation, TCA cycle, glycolysis, and triglyceride synthesis, whereas RIP140 in transgenic mice showed a decreased oxidative capacity activity [163]. RIP140 interacts directly with PGC-1*α* and suppresses its activity [164]. However, RIP140 has also been observed to have a greater effect on PGC-1*β* than PGC-1*α* in adipocytes, probably because of the relatively low levels of PGC-1*α* found in these cells [163]. This may be the case with myogenic cells. C2C12 myogenic cells normally express PGC-1*α* at very low level, whereas PGC-1*β* and PRC were readily detectable under steady-state conditions [72, 92, 165]. Thus, RIP140 suppresses PGC-1*β* and PRC activity more than PGC-1*α*, leading to decreased mitochondrial biogenesis in aged skeletal muscle. Interestingly, ability of RIP140 to repress some mitochondrial genes depends on endogeneous ERR*α* [162], suggesting that the same transcription factor can mediate positive and negative effects on mitochondrial biogenesis depending on the cellular context. PGC-1*α* also contains a negative regulatory domain that attenuates its transcriptional activity, and the p160 myb binding protein (p160^MBP^) acts as a repressor of PGC-1*α* by binding to this regulatory region. This interaction is further regulated by p38 MAPK, which phosphorylates the inhibitory domain of PGC-1*α*, efficiently disrupting p160^MBP^-binding and releasing PGC-1*α* from its inhibition [166]. Adenoviral expression of p160^MBP^ in myoblasts strongly reduced PGC-1*α*'s ability to stimulate mitochondrial respiration and the expression of the genes of the electron transport system [166]. These negative regulators (RIP140 and p160^MBP^) may modulate the activity of PGC-1 family coactivators, leading to decreased mitochondrial biogenesis observed in aged skeletal muscle. There are, however, few reports on expression of these negative regulators in aged skeletal muscle.

## 7. Conclusion

 This paper provides the current knowledge about the molecular mechanisms responsible for age-associated mitochondrial deficiency in skeletal muscle. Several cellular mechanisms would be expected to contribute to mitochondrial deficiency in skeletal muscle. Among them, accumulation of somatic mtDNA mutations appears to correlate with respiratory chain deficiency in aged skeletal muscle. However, the extent to which low level mutation (<1%) can be functionally relevant in ageing is a subject of controversy. The capacity of mitochondrial biogenesis also may be required in maintaining energy production and cellular homeostasis because damaged mitochondria are eliminated during the process of ageing. There is increasing evidence that PGC-1*α* plays a central role in a regulatory network governing the transcriptional control of mitochondrial biogenesis in concert with other transcription factors and nuclear receptors. Intriguingly, it has been demonstrated that transgenic MCK-PGC-1*α* mice have preserved mitochondrial function as well as neuromuscular junctions and muscle integrity during ageing [125]. This suggests that modulation of PGC-1*α* levels in skeletal muscle will offer effective therapeutic potential for prevention and treatment of a group of age-related disorders.
